# Psychological Impact of COVID-19 on University Students in Bahrain

**DOI:** 10.7759/cureus.33079

**Published:** 2022-12-29

**Authors:** Arun Nair, Neha Irfan, Kawther Nemrish, Simone Perna, Dwa Al Khalifa, Amy M Owen-Alkhaldi, Sara Zameer, Maryam Kamali, Ghufran Jassim

**Affiliations:** 1 Department of Pediatrics, Saint Peter’s University Hospital, Somerset, USA; 2 Department of Medicine, Royal College of Surgeons in Ireland - Medical University of Bahrain (RCSI-MUB), Muharraq, BHR; 3 Department of Biology, University of Bahrain, Zallaq, BHR; 4 Center for General Studies, Royal University for Women, Riffa, BHR; 5 Department of Family Medicine, Dubai Health Authority, Dubai, ARE; 6 Department of Family Medicine, Royal College of Surgeons in Ireland - Medical University of Bahrain (RCSI-MUB), Muharraq, BHR

**Keywords:** stigma, anxiety, depression, university students, psychological impact of a pandemic, covid-19

## Abstract

Background

COVID-19 is an ongoing event that has affected millions worldwide financially, socially, and psychologically; university students have also felt the devastating impact. Therefore, assessing how students have been affected psychologically is important, which is explored in this study.

Methods

This cross-sectional study took place in two institutions in Bahrain between October and December 2021. A survey assembled from pre-validated questionnaires was sent through Google Forms after receiving ethics approval. Data were analyzed using network plot analysis and linear regression analysis.

Results

Out of 292 responses, the most common demographic observed were vaccinated Bahraini females living with families at a mean age of 21.21 (SD±3.447). The mean scores for the Generalized Anxiety Disorder Scale (GAD-7), Centered for Epidemiological Studies-Depression scale (CES-D), and Stigma Scale were 10.55 (SD ± 6.144), 18.75 (SD ± 9.147), and 25.40 (SD ± 3.428), respectively. The significant associations on ANOVA observed were stigma score and living status (p-value = 0.002), stigma, and alcohol consumption (p-value = <0.001). Statistically significant predictors of the outcomes in this study included gender for depressive symptoms (p-value* *= 0.013) and alcohol consumption for stigma (p-value = 0.017).

Discussion

The stigmatization of the pandemic may additionally influence sub-threshold depressive and anxiety-related symptoms in university students. Concluding from the results of this study, the psychological impact of the pandemic is significant among university students and needs to be addressed in institutions in Bahrain.

## Introduction

The COVID-19 virus originated in December 2019 and was declared a pandemic by the WHO on January 2020 [1]. The virus is highly transmissible and has posed significant consequences on global health. As of February 2022, over 409 million cases and 5.8 million deaths have been attributed to the COVID-19 virus [2]. People worldwide have been affected in many aspects, including social, economic, cultural, and psychological aspects. As per the guidelines set forth by the Ministry of Health in Bahrain [3], at the peak of the pandemic, restrictions were placed on entering public venues such as malls and recreational areas unless vaccinations were complete. However, as of February 2022, all restrictions were lifted, and current double-dose vaccination rates for the Bahraini population stand at 68% [4]. The constant change in social regulations coupled with the morbid nature of the pandemic can be taxing on the student population. One of the facets to assess the impact of the COVID-19 pandemic is determining the psychological implications of this stressful time. While the pandemic affects every member of the population, some suffer the consequences more gravely, including university students. University students face significant challenges as a consequence of the pandemic. Educational systems have changed drastically, adding stress to academic performance, with financial burdens also contributing to psychological distress [5]. Social isolation, fear, and sensorial deprivation are some products of the pandemic and have been related to anxiety and depression [6]. The pandemic has posed extra stress on the general population, including members involved in the healthcare field, increasing the risk of emotional distress and symptoms of depression and anxiety [7]. Previous studies in the US, Malaysia, and others have shown symptoms of stress, anxiety, and depression among medical and other university students [8, 9].

A meta-analysis conducted by Liyanage S et al. in 2022 suggested that anxiety levels among university students were as high as 41% [10]. Similarly, a cross-sectional study conducted by Wang ZH et al. [11] in a large group of college students in 2020 not only described elevated rates of anxiety and depression, but the need for psychological information and aid had risen to 42% concerning anxiety symptoms and 11.2% for depressive symptoms. One must also consider the stigma individuals may face due to the pandemic. Stigmatization in the era of COVID-19 was investigated by Yuan Y et al. [12], and found that COVID-19 survivors had a higher stigma score, especially concerning rejection in society and internal shame. Mental illness, especially if not addressed adequately and timely, can have substantially long-lasting multifaceted effects on one’s health. Awadalla S et al. in 2020 suggested that around 1/3 of the tertiary student population may be experiencing moderate to severe depression at any given time [13]. There is also a significant association between the presence of depressive symptoms and negative academic performance, which is negated once depression has been treated [13]. Anxiety, however, has had a conflicting effect on an individual’s academic performance, with some studies suggesting better academic performance with increased levels of anxiety and some advocating otherwise. To gain advanced insight into the reasons behind university students’ anxiety, Landejo J in 2021 [14] cited key factors such as self-expectations and fear of disappointing families. Coupling this with the enormous pressurizing circumstances brought upon by the pandemic, anxiety is an essential psychological attribute to be examined and adequately treated, especially among university students. A seldom highlighted factor that may propagate mental illness and the attitude towards it is stigmatization. Existing literature has described the potential social stigma towards the pandemic that has particularly affected the general public’s mental health outcomes, specifically university students. However, the exact risk factors for the development of social stigma are yet to be unraveled [15]. With evidence suggesting mental well-being is crucial, it is essential to investigate the psychological impact of the COVID-19 pandemic on university students. This study aims to examine how university students in Bahrain have been affected psychologically due to the pandemic and, concurrently, examine what risk factors may have contributed to and how institutions may be able to support their students.

## Materials and methods

Study population and demographics

This multicentered cross-sectional study was conducted between October 2021 and December 2021. An electronic survey was sent to students in two universities in Bahrain. The sample population included 292 university students across all the years and disciplines (science, arts, engineering, law, business, and information technology). The inclusion criteria were students who were currently enrolled in at least one course at either university starting October 2021. Participation in the study was entirely voluntary, and participant consent was obtained. The survey was confidential and anonymous, with no identifiers. The study design was reviewed and approved by the ethics review boards of affiliated institutions. The survey was validated in previous studies, and few changes were made to suit the pandemic. The independent variables chosen to investigate the psychological impact included age, gender, nationality, current living arrangements, vaccination status, and questions regarding alcohol and tobacco consumption derived from the Core Alcohol and Drug Survey [16]. The dependent variables chosen included scores for depression, anxiety, and stigma.

Outcome measurement tools

The measurement tools used were (1) the Centered for Epidemiological Studies-Depression scale (CES-D) [17], which was used for depression-associated symptoms, (2) Generalized Anxiety Disorder Scale (GAD-7 scale) [18, 19], which was used to assess all the anxiety-related symptoms, and (3) Stigma Scale [20], which was used to assess perceived stigma against the COVID-19 pandemic.

Centre of Epidemiologic Studies-Depression (CES-D) Scale

The frequency of depressive symptoms among participants was evaluated using the self-administered CES-D scale. Analysis of consistency and validity show the CES-D scale to be a suitable measure of the frequency of depressive symptoms, with its strong psychometric properties [21, 22]. Responses are graded from 0 to 3, with the consensus on total score cut-off at 15 [21], but with variability with score ranges of 0-16, 16-23, and 24-60 indicating mild, moderate, and severe depression, respectively [22].

7-Point Generalized Anxiety Disorder (GAD-7) Scale

The frequency of self-reported anxiety symptoms among participants was evaluated using the GAD-7 scale [23] to assess the frequency of probable anxiety and anxiety-like changes among medical students. The consistency, reliability, and validity of the GAD-7 scale have been successfully tested and confirmed and support the GAD-7 as a good predictive indicator of generalized anxiety [18]. Each question is given a value from 0-3, and a total cut-off score of 10 indicates a recommendation for further evaluation. Scores of 5-9, 10-14, and more than 15 indicate mild, moderate, and severe anxiety, respectively [24, 25].

Stigma Scale

The propensity of perceived stigma around the pandemic was evaluated among medical students by adapting the self-reported stigma scale. The scale is shown to be highly indicative of lived stigma experiences. Each question is analyzed based on the 5-point Likert scale, from strongly disagree to strongly agree [20], with higher scores serving as higher indicators of stigma.

Statistical analysis

Statistical analysis of obtained data was done using the SPSS version 28 for descriptive and inferential analysis. Jeffrey’s Amazing Statistics Program (JASP) was used to generate a network plot analysis to analyze the correlation between both the independent and dependent variables. The significance of inferential statistics is denoted by a p-value less than 0.05. The maximum association coefficient for the JASP association network is 1.

## Results

Demographics

Table 1 demonstrates a descriptive overview of demographics and substance use data. This study collected 292 responses, with different response counts per question due to the voluntary nature of the survey. The mean ± SD age of participants was 21.21 ± 3.447 years. Most participants were female (254 out of 290 (87%)), as one of the institutions this survey was sent to was an all-female tertiary institution. Most respondents lived with family (95%) and received COVID-19 vaccination (90.1%). A total of 92.8% and 67.8% reported no alcohol or tobacco consumption, respectively. Among those who consumed tobacco, E-cigarettes were the most common mode (11%)

**Table 1:** Study population's demographics.

Variables	Categories	N (%)
Age (n=280)	-	21.21 (3.447 +/- 2 SD*) years
Gender (n=290)	Male	36 (12.3%)
	Female	254 (87.0%)
Nationality (n=291)	Bahraini	238 (81.5%)
	Non-Bahraini	53 (18.2%)
Who do you live with (n=291)	Alone	11 (3.8%)
	With a roommate	3 (1.0%)
	With family	277 (94.9%)
Have you received a COVID-19 vaccine? (n=291)	Yes	263 (90.1%)
	No	23 (7.9%)
	Decline to answer	5 (1.7%)
Do you consume alcohol? (n=282)	Yes	11 (3.8%)
	No	271 (92.8%)
Do you consume tobacco? (n=262)	Yes	53 (18.2%)
	No	198 (67.8%)
	Decline to answer	11 (3.8%)
Most preferred method of using tobacco (n=102)	Cigarette	20 (6.8%)
	E-Cigarette	32 (11.0%)
	Shisha (Hookah)	21 (7.2%)
	Other	29 (9.9%)

Descriptive analysis of the scales used

Table 2 describes the associations between the independent variables and the scales used to determine the psychological impact in this study. The average GAD-7 score in this study was found to be 10.55 (SD ± 6.144) on a scale from 0 to 21, and the prevalence of each anxiety category among participants was 22.3% for mild anxiety, 32.2% for moderate, and 25.3% for severe anxiety. No statistically significant relationships were observed between any independent variable and the GAD-7 score.

**Table 2:** Associations between demographic variables and scales scores (ANOVA and t-tests). CESD: Centre for Epidemiological Studies-Depression; GAD-7: Generalized Anxiety Disorder-7.

		*CESD Score, mean (SD)	P-value	**GAD-7 Score, mean (SD)	P-value	Stigma Score, mean (SD)	P-value
Gender	Male	15.000 (8.777)	0.007	9.333 (6.288)	0.182	25.472 (2.286)	0.971
	Female	19.354 (9.045)		10.791 (6.092)		25.492 (3.199)	
Nationality	Bahraini	18.899 (9.119)	0.727	10.546 (6.241)	0.792	25.492 (3.308)	0.998
	Non-Bahraini	18.415 (9.065)		10.792 (5.614)		25.491 (1.857)	
Living status	Alone	16.364 (10.132)	0.300	11.818 (7.346)	0.526	23.000 (6.870)	0.023
	With a family	18.978 (9.067)		10.578 (6.098)		25.596 (2.837)	
	With a roommate	12.333 (6.110)		7.333 (3.215)		25.000 (2.000)	
Vaccination status	Yes	18.905 (9.128)	0.186	10.567 (6.132)	0.065	25.506 (3.201)	0.913
	No	16.522 (8.790)		9.565 (5.711)		25.261 (1.936)	
	Decline to answer	24.400 (6.804)		16.600 (4.930)		25.800 (1.095)	
Alcohol consumption	Yes	19.182 (11.635)	0.925	11.636 (6.376)	0.616	23.909 (6.949)	0.163
	No	18.919 (8.994)		10.705 (6.017)		25.399 (3.260)	
Tobacco consumption	Yes	19.491 (9.551)	0.506	10.962 (6.572)	0.758	25.698 (2.317)	0.341
	No	19.015 (8.922)		10.631 (5.995)		25.717 (2.380)	
	Decline to answer	16.000 (8.602)		9.455 (6.817)		24.636 (2.656)	

On a scale between 0 and 60, the mean score of CES-D was 18.75 (SD ± 9.147), which signifies on average, mild depressive symptoms may be prevalent in this study population. A statistically significant correlation was found between the CES-D score and gender, with a p-value of 0.007. A higher mean CESD-D score was observed among female participants compared to male participants, which was 19.354 (SD ± 9.045). The average stigma score reported was 25.40 (SD ± 3.428) on a scale between 0 and 35, suggesting an elevated level of stigma in this study population towards the pandemic. Table 2 describes a key statistically significant relationship between the stigma score and a participant’s living status, with a p-value of 0.023. Participants who lived with their families were observed to have a slightly higher stigma score of 25.596 (SD ± 2.837).

Regression analysis of the scales used

Table 3 demonstrates a linear regression analysis showing a marginally statistically significant association between tobacco consumption and anxiety levels (p = 0.05) and statistically significant predictability between GAD-7 scores and CES-D scores. This finding supports tobacco consumption as a potentially significant independent predictor of anxiety levels. Of the predictability between the CES-D score and the independent variables of the study, the only statistically significant associations found were between the CES-D score and gender (p = 0.013) and between the CES-D score and GAD-7 score (p = <0.001). This linear regression analysis also revealed a statistically significant association between alcohol consumption and stigma across participants (p = 0.017). The unstandardized B value supports an inverse relationship between the two variables (unstandardized B = 1.900), suggesting that participants who consumed alcohol had lower perceptions of COVID-19-related stigmatization.

**Table 3:** Regression analysis depicting the predictability of the independent variables towards the outcome measures. CES-D: Centre for Epidemiological Studies - Depression; GAD-7: Generalized Anxiety Disorder-7.

	CES-D (adjusted R^2^ = 0.007)		GAD-7 (adjusted R^2^ = 0.029)		Stigma score (adjusted R^2^ = 0.037)	
	Coefficient (95% CI)	P-value	Coefficient (95% CI)	P-value	Coefficient (95% CI)	P-value
Age	0.096 (-0.264 to 0.455)	0.600	-0.047 (-0.291 to 0.197)	0.703	-0.055 (-0.153 to 0.043)	0.267
Gender	4.450 (-0.960 to 7.940)	0.013	1.950 (-0.421 to 4.320)	0.106	-0.071 (-1.021 to 0.878)	0.882
Nationality	0.032 (-2.990 to 3.053)	0.984	0.301 (-1.751 to 2.353)	0.773	0.031 (-0.791 to 0.852)	0.942
Living status	-0.041 (-3.325 to 3.243)	0.980	-1.180 (-3.410 to 1.050)	0.298	0.220 (-0.673 to 1.113)	0.628
Vaccination status	-1.103 (-4.418 to 2.213)	0.513	0.502 (-1.750 to 2.753)	0.661	-0.205 (-1.107 to 0.696)	0.654
Alcohol consumption	1.649 (-4.081 to 7.379)	0.571	0.095 (-3.796 to 3.986)	0.962	1.900 (0.342 to 3.458)	0.017
Tobacco consumption	-2.346 (-5.013 to 0.321)	0.084	-1.812 (-3.623 to -0.001)	0.050	-0.303 (-1.028 to 0.423)	0.412
CES-D score	-	-	1.107 (0.993 to 1.221)	<0.001	0.186 (-0.019 to 0.390)	0.075
GAD-7 score	0.505 (0.453 to 0.557)	<0.001	-	-	-0.023 (-0.161 to 0.116)	0.749
Stigma score	0.059 (-0.006 to 0.124)	0.075	-0.016 (-0.112 to 0.081)	0.749	-	-

## Discussion

In this study, the three key variables assessed to gain an outlook on the psychological impact were depression, anxiety, and stigma. Our results show a significant prevalence of depressive and anxiety-related symptoms across respondents, with gender, tobacco, and alcohol consumption as significant predictors for the CES-D, GAD-7, and stigma scores, respectively. There was a strong correlation between the presence of depressive symptoms and anxiety-like symptoms in participants, as reflected in Table 3, indicating a complex, multifactorial impact of the pandemic on the psychological health of university students. Both anxiety and depressive symptoms had co-dependent predictability with statistically significant p-values (<0.001 in both). This positive correlation is similar to that found in a study conducted by Awano N et al. [26], which also used the GAD-7 and CES-D scales to assess anxiety and depression, respectively. Furthermore, while depressive symptoms were more common among female students (p = 0.007 in Table 2 and p-value = 0.013 in Table 3), the gender of participants was significantly skewed towards females, as one of the participating institutions is a female-only university. This finding can be replicated in a study conducted by Vindegaard N et al. in 2020 [27], who suggested that the female gender was a risk factor for more severe psychiatric symptoms and poorer psychological well-being. This study also reported a good vaccination rate with 90% of participants; however, it is important to recognize vaccine hesitancy in some populations. Although the evidence in the current literature is not vast, it is suggested that individuals with mental illnesses such as anxiety and phobias may have increased hesitancy toward COVID-19 vaccinations [28].

Our study shows a high indication of COVID-19-related stigma across university students in Bahrain, where they expect to receive forms of stigmatization if infected with the virus. A potential confounding factor in this study can be the timing of this study, as the peak of the COVID-19 pandemic, with regard to cases, was observed between April and July 2021 [3]. This study reported an average stigma scale score of 25.4 out of 35, which is significantly elevated and further aligned with a study conducted by Cassiani-Miranda CA et al. [29], who determined a 12.4% prevalence of high stigma discrimination. Interestingly, participants in this study who consumed alcohol showed lower indicators for stigmatization. Interestingly, a study conducted in Poland by Jodczyk AM et al. in 2022 [30] showed a statistically significant association between tobacco and alcohol consumption with a worsened sense of psychological well-being in university students during COVID-19. Although this study reported a statistically significant association between smoking and alcohol use (unstandardized B value was -1.812 and p-value = 0.017 as per Table 3), it is intriguing that alcohol improved a sense of psychological well-being. In contrast, smoking worsened this (unstandardized B value was 1.900 and p-value = 0.050 as per Table 3). As the participant pool in this study predominantly did not consume alcohol or tobacco, it would be recommended to conduct this survey in a larger population with a more balanced proportion of genders. Cultural restrictions on substance use and its open discussion could have also resulted in social acceptability and reporting bias. When comparing rates of depression, anxiety, and stigma in other countries, some similarities are noted. A study conducted by Alkhamees AA et al. among the general population in Saudi Arabia [31] reported the prevalence of moderate to severe depressive and anxiety symptoms being 28.3% and 24%, respectively. This is comparable to this study by using the Impact of Event Scale-Revised (IES-R) and the Depression, Anxiety, and Stress Scale (DASS-21), albeit different scales being used. Comparatively, previous epidemics such as the SARS and H1N1 influenza had anxiety prevalence ranging between 3.2% and 12.6% lower than the COVID-19 pandemic [32]. In this study, 233 out of 292 respondents (79.8%) were classified as having mild, moderate, or severe anxiety, of which one-third were categorized under moderate anxiety (32.2%). This is a significant difference when comparing a similar study using the GAD-7 scale conducted by Zhang Y et al. [33], who reported a 24% overall prevalence of anxiety symptoms in students in China.

In addition to the aforementioned factors that may impact psychological well-being, it is also important to assess factors implicated by social settings and pre-existing psychiatric illnesses. In this study, 32.3% of participants reported GAD-7 scores correlating with moderate anxiety, followed by 25.3% reporting scores for severe anxiety and 22.3% reporting mild anxiety. With the presence of anxiety, symptoms of insomnia and panic disorders may also arise, and the use of benzodiazepines would be an important factor to assess. According to Sarangi A et al., there has been a clinical rise in the diagnosis of insomnia from 14.6% to 20% during the pandemic. In addition, the population above the age of 18 has been reporting increased scores in anxiety screening tools [34]. In correlation with this fact, an increase in the use of benzodiazepines, both prescribed and illegal misuse, has been reported. However, it is a recognizable fact to put into context that this may be a difficult factor to assess in our population due to the taboo nature of the subject. There are three major limitations in this study that could be addressed in further explorations of this topic. Firstly, as one of the institutions involved in this study was an all-women university, gender-associated results are skewed towards a female-predominant sample size, indicating selection bias. Secondly, as noted earlier, cultural considerations may have influenced results relevant to substance use and may serve as a confounding factor that must be considered. Finally, in comparison to other studies that examined the prevalence of depressive, anxiety, and stigma-related manifestations among university students, some discrepancies can be noted. One of the main implications of this observation is the difference in culture, perceived information regarding the pandemic through the different media outlets in different countries, as well as the difference in curriculum and living situations among students. Therefore, larger sample sizes and a randomized sample for this study may have provided more accurate results and decreased the margin of error in this study and thereby would have allowed for more precise examinations of the complex interactions of the variables involved.

## Conclusions

This study brings essential intel on the psychological health and well-being of university students in Bahrain during the era of COVID-19. Understanding the mental health status of university students, particularly during such unprecedented times, is crucial to developing mechanisms to mitigate short- and long-term consequences. For instance, a better understanding of different factors that affect students’ during this pandemic can help institutions cater to high-risk individuals when customizing mental health support through individualized counseling sessions and support groups. Further studies are needed to analyze the most appropriate therapy and support sessions considering the cultural implications in respective countries. This would reveal effective means of honing mental health and wellness in future generations of professionals.
